# Predictive value of Gd-IgA1, poly-IgA in the treatment of IgA nephropathy with targeted-release formulation budesonide

**DOI:** 10.1093/ckj/sfaf203

**Published:** 2025-07-01

**Authors:** Qinlan Chen, Pei Chen, Rong He, Jincan Zan, Xue Shen, Jicheng Lv, Hong Zhang

**Affiliations:** Renal Division, Department of Medicine, Peking University First Hospital, Beijing China; Institute of Nephrology, Peking University, Beiling, China; Key Laboratory of Renal Disease, Ministry of Health of China, Beijing, China; Key Laboratory of Chronic Kidney Disease Prevention and Treatment (Peking University), Ministry of Education, Beijing 100034, China; Renal Division, Department of Medicine, Peking University First Hospital, Beijing China; Institute of Nephrology, Peking University, Beiling, China; Key Laboratory of Renal Disease, Ministry of Health of China, Beijing, China; Key Laboratory of Chronic Kidney Disease Prevention and Treatment (Peking University), Ministry of Education, Beijing 100034, China; Renal Division, Department of Medicine, Peking University First Hospital, Beijing China; Institute of Nephrology, Peking University, Beiling, China; Key Laboratory of Renal Disease, Ministry of Health of China, Beijing, China; Key Laboratory of Chronic Kidney Disease Prevention and Treatment (Peking University), Ministry of Education, Beijing 100034, China; Department of Nephrology, Guizhou Provincial People’s Hospital, National Health Commission Key Laboratory of Pulmonary Immunological Diseases, Guiyang, China; Renal Division, Department of Medicine, Peking University First Hospital, Beijing China; Institute of Nephrology, Peking University, Beiling, China; Key Laboratory of Renal Disease, Ministry of Health of China, Beijing, China; Key Laboratory of Chronic Kidney Disease Prevention and Treatment (Peking University), Ministry of Education, Beijing 100034, China; Renal Division, Department of Medicine, Peking University First Hospital, Beijing China; Institute of Nephrology, Peking University, Beiling, China; Key Laboratory of Renal Disease, Ministry of Health of China, Beijing, China; Key Laboratory of Chronic Kidney Disease Prevention and Treatment (Peking University), Ministry of Education, Beijing 100034, China; Renal Division, Department of Medicine, Peking University First Hospital, Beijing China; Institute of Nephrology, Peking University, Beiling, China; Key Laboratory of Renal Disease, Ministry of Health of China, Beijing, China; Key Laboratory of Chronic Kidney Disease Prevention and Treatment (Peking University), Ministry of Education, Beijing 100034, China; Renal Division, Department of Medicine, Peking University First Hospital, Beijing China; Institute of Nephrology, Peking University, Beiling, China; Key Laboratory of Renal Disease, Ministry of Health of China, Beijing, China; Key Laboratory of Chronic Kidney Disease Prevention and Treatment (Peking University), Ministry of Education, Beijing 100034, China

**Keywords:** Gd-IgA1, IgA nephropathy, Nefecon, poly-IgA, proteinuria

## Abstract

**Background:**

Targeted release formulation (TRF) budesonide (Nefecon), targeting galactose-deficient immunoglobulin A1 (Gd-IgA1) production and IgA immune complex formation, has been approved for IgA nephropathy (IgAN) treatment. In this study we explored whether early changes in these biomarkers can predict the clinical response to Nefecon therapy.

**Methods:**

Plasma samples from 27 IgAN patients treated with Nefecon and followed at least 6 months were collected during routine visits. We measured the levels of Gd-IgA1 and poly-IgA during the treatment, analysing the association between their baseline levels or changes and proteinuria reduction.

**Results:**

The mean proteinuria level was 1.3 ± 0.8 g/day and the estimated glomerular filtration rate was 47.1 ± 21.7 ml/min/1.73 m^2^ at baseline. During the follow-up, proteinuria slowly decreased, with alterations of −0.12 g/day, −0.42 g/day, −0.58 g/day and −0.86 g/day at 3, 6, 9 and 12 months, respectively. The plasma levels of Gd-IgA1, poly-IgA and total IgA decreased after Nefecon treatment, with an obvious decrease at 2 months in Gd-IgA1 by −1067.3 ng/ml and poly-IgA by −1.18 mg/l. All biomarker reductions were strongly associated with a proteinuria decrease (*P* < .0001). Importantly, the early reduction in poly-IgA during the first 2 months was associated with a proteinuria reduction at 6 months (*R* = 0.47, *P* = .01). Similar trends were observed for Gd-IgA1, though not statistically significant.

**Conclusions:**

The early changes in Gd-IgA1 or poly-IgA, especially poly-IgA, were associated with future proteinuria reduction, supporting the potential of Gd-IgA1 and poly-IgA as biomarkers for predicting Nefecon response in IgAN.

KEY LEARNING POINTS **What was known:**

- Targeted-release formulation (TRF) budesonide (Nefecon), targeting nephritogenic immunoglobulin A1 (IgA1) production, has demonstrated clear kidney benefits for IgA nephropathy (IgAN).
- To date, no validated plasma or urine biomarkers are available to guide treatment or predict response of Nefecon in patients with IgAN.

**This study adds:**

- We evaluated the association between galactose-deficient IgA1 (Gd-IgA1) or poly-IgA and proteinuria reduction and determined whether baseline or early reductions in these biomarkers can predict long-term clinical response in patients treated with Nefecon.
- Our study showed a strong association between the reductions in Gd-IgA1 and poly-IgA, particularly poly-IgA, and the reduction in proteinuria. Notably, we observed that early reductions in poly-IgA in the first 2 months demonstrated a significant correlation with proteinuria reduction at 6 months.

**Potential impact:**

- The forms of IgA and IgA immune complexes, including Gd-IgA1 and poly-IgA immune complex, may serve as biomarkers to guide TRF budesonide treatment.
- The findings still need to be validated in large, prospective multicentre cohort studies.

## INTRODUCTION

Immunoglobulin A nephropathy (IgAN), also referred to as Berger's disease, is the most common form of biopsy-confirmed primary glomerular disease [1]. Epidemiological evidence from a large UK cohort study demonstrated that a significant proportion of patients progressed to end-stage kidney disease (ESKD) within 10–15 years of diagnosis, indicating potentially worse long-term prognosis than previously anticipated [2]. There is an urgent need for safe and effective novel treatments to enhance the outcomes of IgAN.

The pathogenesis of IgAN provides insights for the development of novel therapeutic approaches. The ‘multihit’ hypothesis offers a comprehensive framework for understanding the pathogenesis of IgAN. Circulating galactose-deficient IgA1 (Gd-IgA1), predominantly originating from mucosal immune responses, is recognized by circulating IgG or IgA autoantibodies, ultimately leading to the formation of pathogenic poly-IgA immune complexes, which are considered the source of glomerular mesangial deposition of IgA1 immune complexes, and consists of mainly polymeric IgA, along with IgG, IgM and complement components. CD89 is a type I transmembrane glycoprotein expressed on the surface of myeloid cells and binds both IgA1 and IgA2 molecules through its amino terminus Ig domain that interacts with the Cα2/Cα3 junction of IgA. It has been demonstrated that CD89 exhibits higher affinity to poly-IgA than to mono-IgA, allowing phagocytes to selectively capture poly-IgA complexes [3]. These complexes are subsequently deposited in the glomerular mesangium, initiating mesangial cell activation and subsequent glomerular injury [4–6]. Consequently, therapeutic strategies aimed at reducing or preventing the formation of IgA immune complexes have emerged as a central focus in IgAN management.

Targeted release formulation (TRF) budesonide (Nefecon), an orally administered glucocorticoid, has been specifically engineered to selectively target the ileal gut-associated lymphoid tissue. A linear regression model was used to extrapolate the effect of Nefecon on the estimated glomerular filtration rate (eGFR) slope in the NefIgArd trial (NCT03643965) and it indicates that Nefecon may delay progression to the

clinical outcome of kidney failure, eGFR <15 ml/min/1.73 m^2^ or sustained doubling of serum creatinine by ≈12.8 years compared with supportive care alone [7]. The NefIgArd trial demonstrated that Nefecon treatment significantly reduced proteinuria in patients with IgAN, with the maximal therapeutic effect typically observed at 12 months, following an initial latency period of at least 3 months [8]. Notably, this proteinuria-lowering effect remained consistent even in patients with severe renal impairment (eGFR 25–35 ml/min/1.73 m^2^) [9]. Despite these promising results, the absence of validated plasma or urine biomarkers for Nefecon treatment guidance and response prediction poses a significant challenge in optimizing individualized therapeutic strategies. While the phase 2b NEFIGAN trial (NCT01738035) demonstrated significant reductions in serum Gd-IgA1 and IgA–IgG immune complexes in response to Nefecon treatment [10, 11], critical questions remain unanswered. Specifically, whether early changes or baseline levels of Gd-IgA1 or polymeric IgA forms can predict long-term therapeutic response have not been systematically investigated.

This study aims to investigate the correlation between changes in Gd-IgA1 or poly-IgA and proteinuria reduction, while evaluating whether baseline measurements or early biomarker changes can predict long-term clinical response in patients treated with Nefecon.

## MATERIALS AND METHODS

### Study protocol and participants

We conducted an observational study in adult patients with biopsy-confirmed IgAN. Eligible participants had a baseline proteinuria level >0.5 g/day and an eGFR ≥20 ml/min/1.73 m^2^ and were treated with Nefecon. All patients were followed for at least 6 months after the initiation of Nefecon treatment. Sequential plasma samples were collected and retained at regular clinic follow-up visits between May 2023 and February 2025. Blood samples were centrifuged immediately after collection and stored at −80°C until analysis. Demographic and clinical data were recorded for all patients. The study was approved by the local ethics committee (2024-482).

### Measurement of biomarkers by enzyme-linked immunosorbent assay (ELISA)

Plasma Gd-IgA1 concentrations were evaluated using an ELISA kit precoated with KM55 (Immuno-Biological Laboratories, Fujioka-Shi, Japan). Briefly, the ELISA plates were incubated with plasma specimens diluted 200-fold with enzyme immunoassay buffer and other procedures were carried out according to the manufacturer's instructions. Gd-IgA1 concentrations were quantified using a standard curve generated from known concentrations of the analyte. Poly-IgA levels were measured as described previously [12]. Briefly, ELISA plates were coated with recombinant CD89 at a concentration of 2.5 μg/ml. Samples were diluted 1000-fold with phosphate-buffered saline with Tween/1% bovine serum albumin. Following the incubation and washing steps, antihuman IgA-HRP detection antibody was added at a 1:20 000 dilution. The reaction was developed by adding tetramethylbenzidine liquid substrate system. The poly-IgA concentrations were evaluated according to the standard curve. The total IgA level was detected by ELISA using a F(ab′)2 fragment of goat anti-human IgA (Jackson ImmunoResearch, West Grove, PA, USA) as the capture molecular probe and goat anti-human IgA alpha chain (HRP) pre-adsorbed (ab98558; Abcam, Cambridge, UK) as the detection antibody. Cross-capture ELISA was used to measure plasma IgA–IgG immune complex levels. A F(ab′)2 fragment of goat anti-human IgA (Jackson ImmunoResearch) was used as the capture antibody and anti- human IgG antibody (ab200699; Abcam) was used as the detection antibody.

### Statistical analysis

All missing data were treated as missing without imputation. Log_10_ transformations were applied to biomarkers and urine protein to meet model distributional assumptions. Depending on the distribution of the variables, either Spearman or Pearson correlation coefficients were calculated between the log transformed biomarkers and urine protein. Differences in means for continuous variables between two groups were compared using the *t*-test. Non-normally distributed data were compared using the non-parametric Mann–Whitney U test. All statistical analyses were performed using SPSS version 26 (IBM, Armonk, NY, USA) and GraphPad Prism version 10.1.2 (GraphPad Software, Boston, MA, USA). *P*-values <.05 were considered statistically significant.

## RESULTS

### Demographic information and clinical characteristics of participants

**Figure 1:**
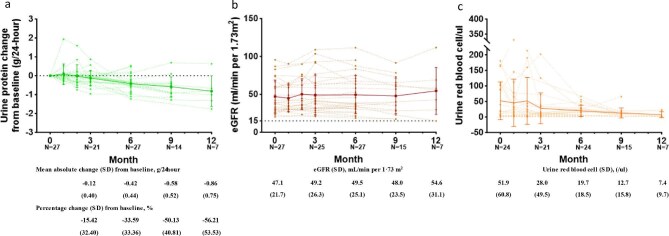
Trends in **(a)** urine protein, **(b)** eGFR and **(c)** haematuria for patients treated with Nefecon. The dashed lines represent the individual patient trajectories, whereas the solid lines indicate the average changes in (a) urine protein, (b) eGFR and (c) haematuria.

There were 27 patients enrolled in this study. All patients had biopsy-confirmed IgAN, with a mean duration of 64.2 ± 57.9 months since kidney biopsy. Seventeen were male, with a mean age of 41 years (range 27–62). All the patients received supportive therapy that included renin–angiotensin system (RAS) blockade and 37% of them had previously received immunosuppressive therapy. The dosage of concomitant medication remained stable throughout the course of Nefecon treatment. Baseline clinical parameters included a mean proteinuria level of 1.3 ± 0.8 g/day and a mean eGFR of 47.1 ± 21.7 ml/min/1.73 m^2^. Additionally, 10 of the 27 patients were found to have microhaematuria. Comprehensive baseline characteristics are detailed in Table 1. Following Nefecon treatment, proteinuria levels demonstrated a progressive reduction from baseline, with mean changes of −0.12 ± 0.40 g/day at 3 months, −0.42 ± 0.44 g/day at 6 months, −0.58 ± 0.52 g/day at 9 months and −0.86 ± 0.75 g/day at 12 months (Fig. 1a). The therapeutic response exhibited a gradual pattern, with a less pronounced reduction during the initial 3-month period. Nine patients (33.3%) achieved complete remission, defined as a proteinuria level <0.3 g/day. During the follow-up period, only 1 patient (3.7%) progressed to ESKD, while the remaining 26 patients (96.3%) maintained stable renal function. Notably, the cohort demonstrated a mean improvement in eGFR of 7.52 ml/min/1.73 m^2^ (Fig. 1b). Nefecon treatment also led to a gradual decrease in urine red blood cells, with mean levels of 51.9 ± 60.8/μl at baseline, 28.0 ± 49.5/μl at 3 months, 19.7 ± 18.5/μl at 6 months, 12.7 ± 15.8/μl at 9 months and 7.4 ± 9.7/μl at 12 months (Fig. 1c). Haematuria was observed in 42% (10/24) of patients at baseline, decreasing to 21% at 3 months and increasing slightly to 33% at 6 months. No adverse events leading to death were reported and a comprehensive listing of adverse events is presented in Supplementary data Table S1.

**Table 1:** Main clinical and laboratory characteristics at baseline.

Characteristics	Values
Male, *n* (%)	17 (63.0)
Age (years), mean ± SD	41.30 ± 9.18
BMI (kg/m^2^), mean ± SD	23.70 ± 3.39
Previous use of systemic glucocorticoids or immunosuppressants, *n* (%)	10 (37.0)
Concomitant medications, *n* (%)	
RASI	27 (100)
SGLT2i	15 (55.6)
ERA	19 (70.4)
MRA	11 (40.7)
HCQ	8 (29.6)
TwHF	14 (51.9)
Blood pressure (mmHg), mean ± SD	
Systolic	116.00 ± 10.96
Diastolic	75.70 ± 9.08
Urine protein (g/day), mean ± SD)	1.30 ± 0.80
eGFR (ml/min/1.73 m^2^), mean ± SD	47.05 ± 21.71
Serum albumin (g/l), mean ± SD	38.41 ± 4.11
Haematuria (RBCs/μl), mean ± SD	51.88 ± 60.80
Immunoglobulins, mean ± SD	
Gd-IgA1 (ng/ml)	4895.06 ± 2168.76
Poly-IgA (mg/l)	8.46 ± 4.38
Total IgA (g/l)	2.35 ± 0.66
IgA–IgG immune complex (mg/l)	421.05 ± 100.16
Time since kidney biopsy (months), mean ± SD	64.2 ± 57.9
Treatment duration of Nefecon (months), mean ± SD	8.0 ± 2.0
Oxford classification, *n* (%)	
M1	23 (85.2)
E1	14 (51.9)
S1	21 (87.5)
T1 + T2	13 (54.2)
C1 + C2	17 (70.8)

BMI: body mass index; C: crescents; E: endocapillary hypercellularity; ERA: endothelin receptor antagonist; HCQ: hydroxychloroquine; M: mesangial hypercellularity; MRA: mineralocorticoid receptor antagonist; RASI: renin–angiotensin system inhibitor; RBC: red blood cell; S: segmental glomerulosclerosis or adhesion; SGLT2i: sodium–glucose co-transporter 2 inhibitor; T: tubular atrophy/interstitial fibrosis; TwHF: *Tripterygium wilfordii* Hook. F.

### Change in the biomarkers after Nefecon treatment

**Figure 2:**
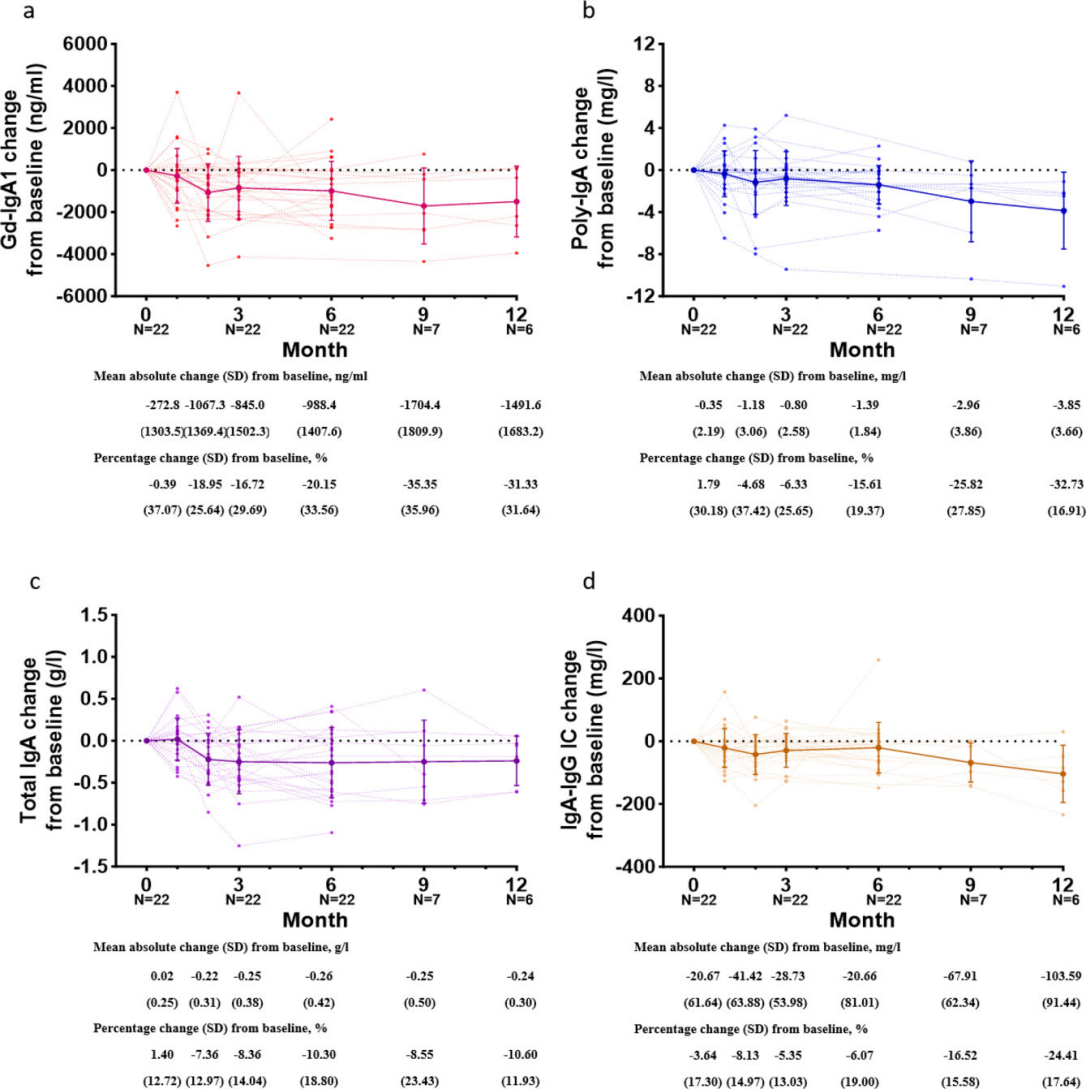
Effects of Nefecon on biomarkers. Absolute changes from baseline at each visit in **(a)** Gd-IgA1, **(b)** poly-IgA, **(c)** total IgA and **(d)** IgA–IgG immune complex. IC: immune complex.

**Figure 3:**
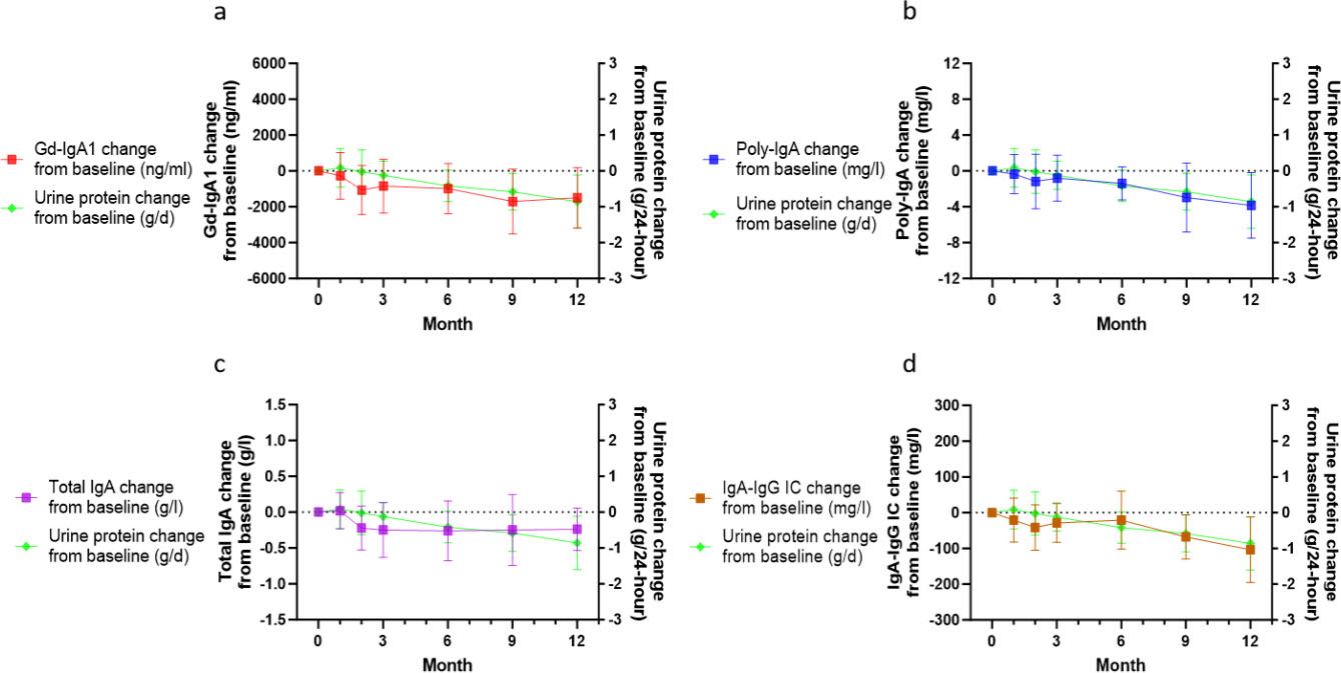
Effects of Nefecon on biomarkers and urine protein. Absolute changes from baseline at each visit in **(a)** Gd-IgA1 and urine protein, **(b)** poly-IgA and urine protein, **(c)** total IgA and urine protein and **(d)** IgA–IgG immune complex and urine protein. IC: immune complex.

The trajectories of these biomarker changes following Nefecon treatment are illustrated in Fig. 2. Progressive reductions were observed in Gd-IgA1, poly-IgA total IgA and IgA–IgG immune complex levels following treatment initiation. An obvious decrease was observed at 2 months after Nefecon initiation, with a mean alteration in Gd-IgA1 of −1067.3 ± 1369.4 ng/ml, in poly-IgA of −1.18 ± 3.06 mg/l, in total IgA of −0.22 ± 0.31 g/l and in IgA–IgG immune complex of −41.42 ± 63.88 mg/l. Parallel trends were observed between the biomarkers and urine protein levels (Fig. 3), prompting further investigation of their statistical correlations.

### Association of biomarkers and proteinuria reduction

**Figure 4:**
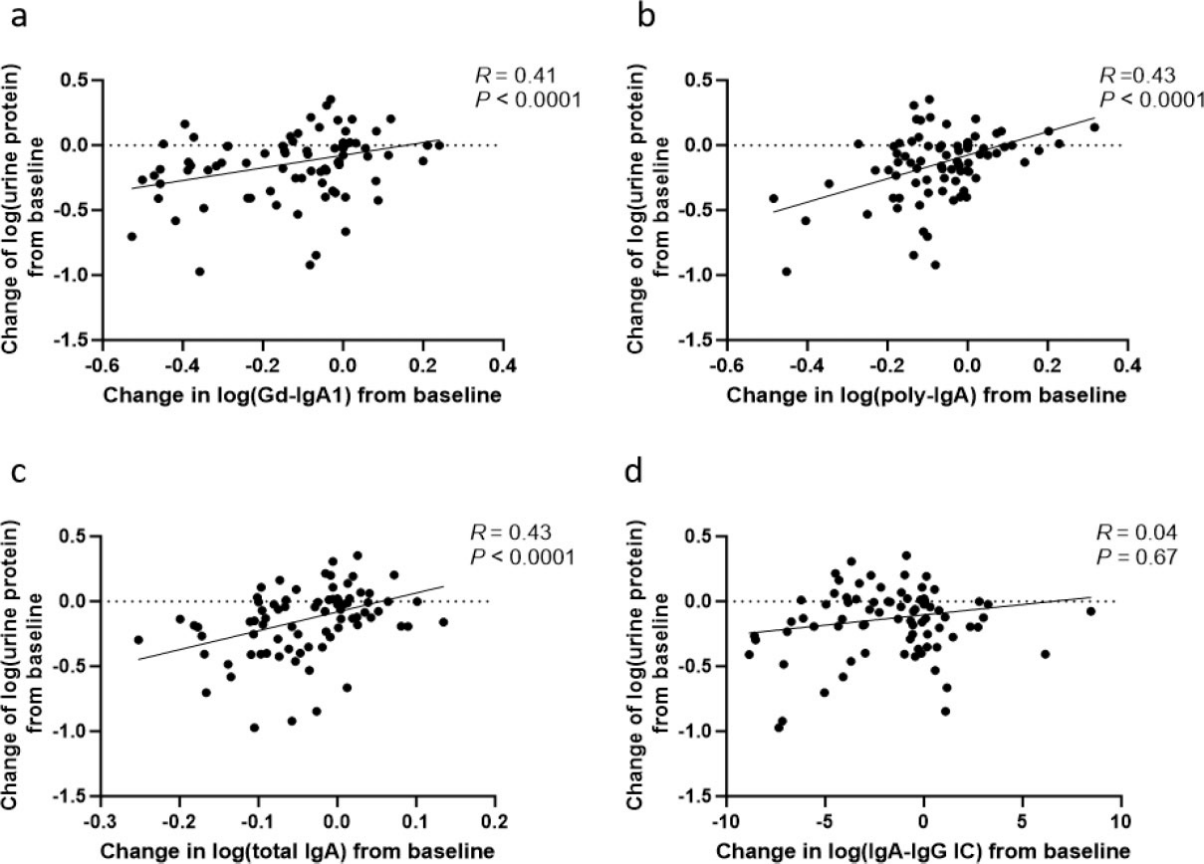
The correlation between the changes of biomarkers and proteinuria over time throughout the study. Scatter plots showing the association between the changes in proteinuria from baseline and the changes in **(a)** Gd-IgA1, **(b)** poly-IgA, **(c)** total IgA and **(d)** IgA–IgG immune complex from baseline. IC: immune complex.

**Figure 5:**
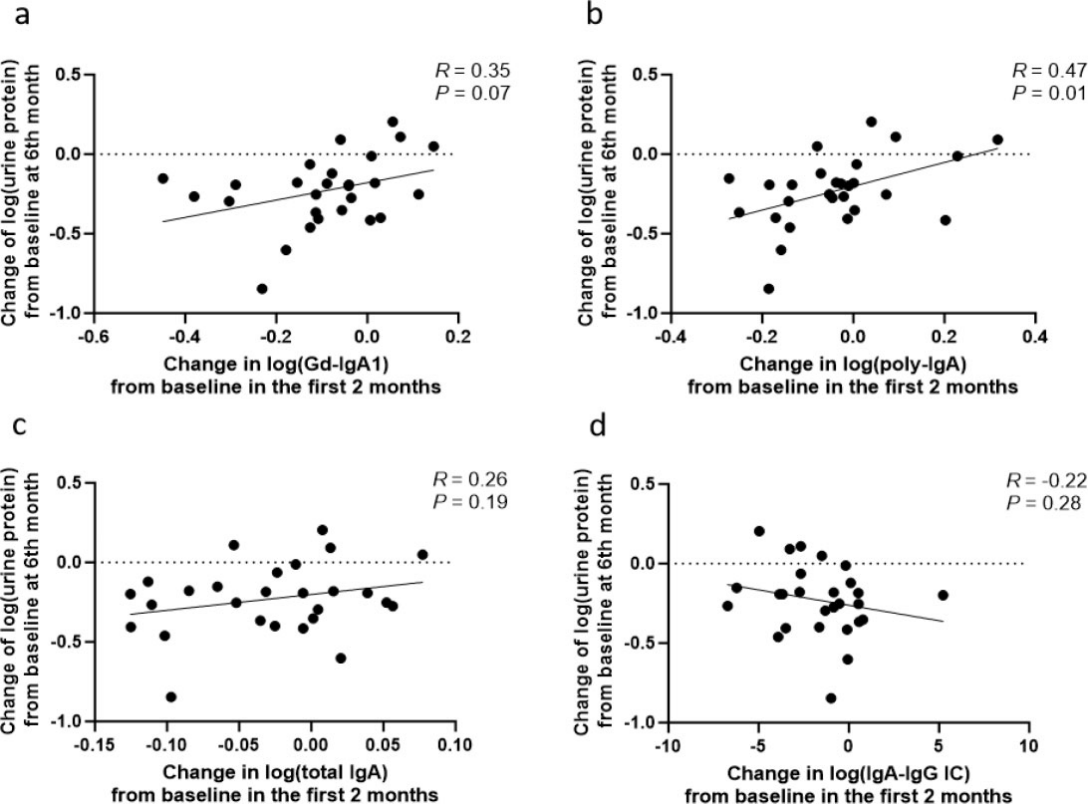
The correlation between the changes in biomarkers within the first 2 months and the changes in proteinuria at 6 months. Scatter plots showing the association between the changes in proteinuria at 6 months and the changes in **(a)** Gd-IgA1, **(b)** poly-IgA, **(c)** total IgA and **(d)** IgA–IgG immune complex from baseline within the first 2 months of treatment. IC: immune complex.

**Figure 6:**
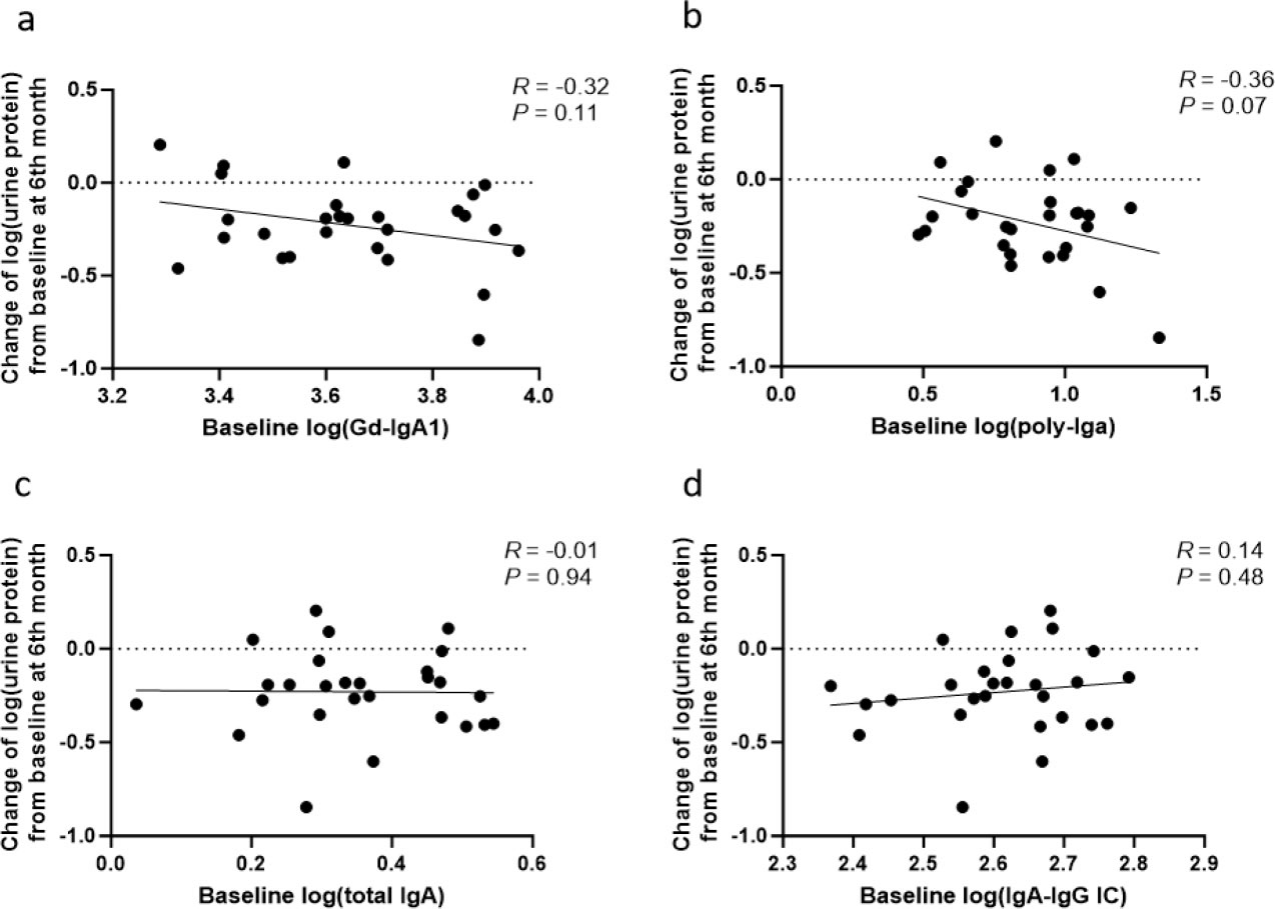
The correlation between baseline biomarkers and the changes in proteinuria at 6 months. Scatter plots showing the association between the changes in proteinuria from baseline at 6 months and baseline levels of **(a)** Gd-IgA1, **(b)** poly-IgA, **(c)** total IgA and **(d)** IgA–IgG immune complex. IC: immune complex.

**Figure 7:**
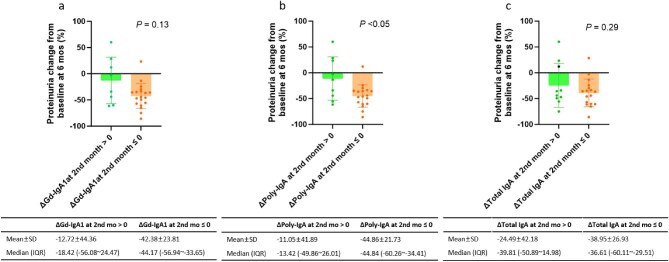
Effects of Nefecon on proteinuria changes in patient groups stratified by early biomarker responses. Changes in proteinuria at 6 months were compared between groups with elevated or reduced levels of **(a)** Gd-IgA1, **(b)** poly-IgA and **(c)** total IgA at 2 months.

Throughout the treatment period, changes in proteinuria showed a positive correlation with Gd-IgA1, poly-IgA and total IgA from baseline, all of which reached statistical significance. In contrast, no significant association was observed between changes in the IgA–IgG immune complex and proteinuria (*R* = 0.04, *P* = .67; Fig. 4). Extending prior observations of the Gd-IgA1–proteinuria association, our analysis revealed that early (2-month) reductions in poly-IgA (*R* = 0.47, *P* = .01; Fig. 5b) and Gd-IgA1 (*R* = 0.35, *P* = .07; Fig. 5a) were positively correlated with proteinuria reduction at 6 months. Notably, poly-IgA demonstrated superior predictive capacity compared with other biomarkers. While total IgA showed a similar but weaker trend (*R* = 0.26, *P* = .19; Fig. 5c), IgA–IgG immune complex levels exhibited no clinically meaningful association with proteinuria reduction (*R* = −0.22, *P* = .28; Fig. 5d). Additionally, patients with higher baseline levels of poly-IgA (*R* = −0.36, *P* = .07; Fig. 6b), Gd-IgA1 (*R* = −0.32, *P* = .11; Fig. 6a) or total IgA (*R* = −0.01, *P* = .94; Fig. 6c) showed a tendency toward greater proteinuria reduction compared with those with lower baseline levels, although this trend was not statistically significant, suggesting that pretreatment biomarker profiling may help identify patients with a better therapeutic potential for Nefecon. No meaningful correlation was observed between baseline plasma IgA–IgG changes and later proteinuria reduction (*R* = 0.14, *P* = .48; Fig. 6d). We also observed that patients exhibiting a reduction in biomarker levels at 2 months tended to achieve a greater proteinuria reduction at 6 months compared with those with increased biomarker levels. However, only the association for poly-IgA reached statistical significance (Fig. 7).

## DISCUSSION

Therapeutic strategies targeting pathogenic IgA1 production, such as TRF budesonide, have demonstrated significant renal protective effects for IgAN, manifesting as a substantial proteinuria reduction and attenuation of eGFR decline. Consistent with these clinical benefits, treatment with TRF budesonide has been associated with reductions in serum levels of key immunological markers, including B cell activation factor (BAFF), a proliferation-inducing ligand (APRIL), Gd-IgA1, secretory IgA and IgA–IgG immune complexes [10, 13]. In this study, we first explored the correlation between Gd-IgA, poly-IgA, total IgA and proteinuria reductions while evaluating the predictive value of baseline levels and early biomarker reductions for proteinuria reduction during TRF budesonide treatment. Our results confirmed a strong association between a reduction in Gd-IgA1, poly-IgA and total IgA and proteinuria. Notably, early reduction in poly-IgA levels during the initial 2 months of treatment demonstrated a significant correlation with proteinuria reduction at 6 months, and patients exhibiting a reduction in three biomarker levels at 2 months tended to achieve a greater proteinuria reduction at 6 months compared with those with increased biomarker levels. While similar positive trends were observed for Gd-IgA1 and total IgA, these correlations did not achieve statistical significance, possibly due to the small sample size. We also observed that the proportions of Gd-IgA1 and poly-IgA within total IgA decreased significantly during the first 2 months, followed by a rebound after 9 months (Supplementary data Fig. S2). These findings align with the proposed mechanism of Nefecon action, which involves a reduction of pathogenic IgA forms and IgA immune complexes. Furthermore, beyond confirming the known link between Gd-IgA1 reduction and proteinuria improvement, our data newly identify poly-IgA as a potential superior predictor as well as the clinical relevance of early biomarker changes. These findings may guide treatment with TRF budesonide in clinical practice.

The prevention and reduction of pathogenic IgA immune complex formation has emerged as a crucial therapeutic target in IgAN management. Recent therapeutic advancements have focused on multiple pathogenic pathways, including B cell activation in gut-associated lymphoid tissue (GALT), modulation of APRIL and BAFF cytokine pathways and plasma cell regulation. Clinical evidence from previous studies demonstrated significant reductions in serum Gd-IgA1 levels following Nefecon treatment (16 mg/day), with reductions of 21.4% at 3 months, 23.5% at 6 months and 34% at 9 months [14]. Our findings corroborate these observations, showing 20.2% and 35.4% reductions in Gd-IgA1 levels at 6 and 9 months, respectively, consistent with data from the phase 3 NefIgArd trial. The NefIgArd trial demonstrated a delayed therapeutic response, with proteinuria reduction becoming evident only after 3 months of treatment, reaching 17% at 6 months and 30% at 9 months compared with placebo [8]. The time lag between biomarker alterations and proteinuria changes may reflect Nefecon's unique mechanism of action, which involves selective targeting of GALT—the proposed upstream pathogenic site according to the ‘multiple-hit hypothesis’—with subsequent downstream renal protective effects manifesting months later [15]. Notably, our study cohort exhibited more advanced disease characteristics compared with the NefIgArd trial population, with lower baseline eGFR (including six patients with eGFR <30 ml/min/1.73 m^2^). Given the established association between elevated poly-IgA immune complex levels and disease severity or treatment responsiveness to steroids and telitacicept [3, 12], baseline poly-IgA levels or their early reduction may serve as predictive biomarkers for long-term clinical response to therapies targeting pathogenic IgA in IgAN.

Additionally, while changes in all three biomarkers demonstrated significant associations with proteinuria alterations, baseline circulating poly-IgA levels and their early changes within the initial 2 months exhibited stronger correlations with proteinuria reduction at 6 months. Considering the established pathogenic roles of Gd-IgA1 and poly-IgA in IgAN, we propose that poly-IgA complexes, rather than isolated Gd-IgA1 molecules, constitute the primary source of renal immune deposits. This superior predictive performance of poly-IgA is further supported by this study and our previous findings demonstrating its correlation with treatment response to telitacicept [12], suggesting its potential as a clinically useful biomarker across different therapeutic approaches in IgAN management. This hypothesis is supported by clinical observations that elevated circulating Gd-IgA1 levels alone are insufficient to induce IgAN, as evidenced by asymptomatic first-degree relatives of IgAN patients who maintain high Gd-IgA1 levels [16, 17]. The propensity of serum Gd-IgA1 to form self-aggregated poly-IgA complexes is well-documented, consistent with its predominant presence within high molecular weight immune complexes [17–19]. Importantly, poly-IgA complexes containing Gd-IgA1 demonstrate significantly enhanced binding affinity to mesangial cells compared with uncomplexed IgA1 in IgAN patients. In addition, uncomplexed IgA1 failed to induce cellular proliferation, whereas native serum IgA1 complexes from IgAN patients enhanced cellular proliferation [19, 20]. Our previous research has established a correlation between elevated poly-IgA immune complex levels and both disease severity and therapeutic response to steroids and immunosuppressants [3]. The lack of correlation between total IgA and proteinuria, in contrast to poly-IgA or Gd-IgA1, may be explained by its composite nature, as circulating total IgA encompasses both mono-IgA and poly-IgA forms, potentially diluting both pathogenic signals.

Interestingly, we observed differential biomarker responses following Nefecon discontinuation, with Gd-IgA1 levels rebounding 3 months after treatment cessation while poly-IgA levels remained suppressed. This observation aligns with findings from the phase 3 NefIgArd trial, which documented proteinuria recurrence and eGFR decline following treatment discontinuation [8]. These findings suggest that sustained disease control may require either repeated 9-month Nefecon cycles or implementation of a reduced-dose maintenance regimen. A particularly instructive case was Patient 10, who maintained persistent proteinuria (>0.5 g/day) following Nefecon completion. This patient subsequently initiated telitacicept therapy 1 month later, with subsequent biomarker and clinical data excluded from our primary analysis. Notably, this patient demonstrated further reductions in poly-IgA, Gd-IgA1 and total IgA levels following telitacicept initiation, accompanied by a complete proteinuria remission (Supplementary data Fig. S1). This synergistic effect suggests potential therapeutic benefits from combination therapy targeting IgA immune complex formation, although this approach warrants further systematic investigation. In addition, consistent with the dose-dependent relationship observed for poly-IgA and proteinuria reduction in telitacicept trials, patients receiving Nefecon 16 mg demonstrated significantly greater reductions in both Gd-IgA1 levels and proteinuria compared with those treated with the 8 mg dose [10, 21]. These findings suggest a possible dose-dependent effect of Nefecon on poly-IgA reduction, which may inform optimal therapeutic strategies and dose titration protocols, although this requires validation through systematic investigation.

Our study is the first to evaluate the association between Gd-IgA1 or poly-IgA and proteinuria reduction in patients receiving Nefecon therapy. However, there are several key limitations to this study. First, this is a case series with just 27 participants, limiting the study's ability to detect significant associations between biomarkers and proteinuria reduction. Second, this is an observational study and not all patients provided plasma samples at each visit. Third, the maximum follow-up duration was only 1 year, limiting the study's ability to assess the long-term association between biomarkers and kidney progression. Overall, the findings need to be validated in large, prospective multicentre cohort studies.

Collectively, the data suggest that forms of IgA and IgA immune complexes, including Gd-IgA1 and poly-IgA, especially poly-IgA, may serve as biomarkers to guide TRF budesonide treatment. However, this needs to be confirmed in large, prospective cohort studies.

## Supplementary Material

sfaf203_Supplemental_File

## Data Availability

The data underlying this article will be shared upon reasonable request to the corresponding author.
