# Supplementary material for: Predictive value of Gd-IgA1, poly-IgA in the treatment of IgA nephropathy with targeted-release formulation budesonide
Source: Clin Kidney J. 2025 Jul 1;18(7):sfaf203. doi: 10.1093/ckj/sfaf203 (PMC12268327; doi:10.1093/ckj/sfaf203)
Supplement: sfaf203_Supplemental_File [file sfaf203_supplemental_file.pdf]

Supplemental Table S1. Adverse events during Nefecon treatment period.

Adverse events	Number of patients with event (n, %)
Increase in weight	15 (55.5%)
Upper respiratory tract infection	10 (37.0%)
Hypertension	8 (29.6%)
Rash	7 (25.9%)
Face oedema	7 (25.9%)
Acne	6 (22.2%)
Peripheral oedema	5 (18.5%)
Muscle spasms	3 (11.1%)
Pneumonia	2 (7.4%)
Impaired glucose tolerance	2 (7.4%)
Insomnia	2 (7.4%)
Urinary tract infection	2 (7.4%)
Other infection	2 (7.4%)
Rapid hair growth	2 (7.4%)
Diabetes*	1 (3.7%)
Fatigue	1 (3.7%)
Arthralgia	1 (3.7%)
Headache	1 (3.7%)
Acute pancreatitis*	1 (3.7%)
Impaired vision	1 (3.7%)
Lipsotrichia	1 (3.7%)
Menstrual disorder	1 (3.7%)
Increase in bilirubin	1 (3.7%)
Purple striae	1 (3.7%)
Palpitation	1 (3.7%)
Aphthous stomatitis	1 (3.7%)
Gingival atrophy	1 (3.7%)
Acid regurgitation	1 (3.7%)
Buffalo hump	1 (3.7%)

*Includes patients received hospitalization

Supplemental Figure S1. Changes of Patient 10 in Gd-IgA1, poly-IgA, total IgA, and proteinuria from baseline. Red arrow: End of Nefecon treatment; Blue arrow: Time of telitacicept initiation.

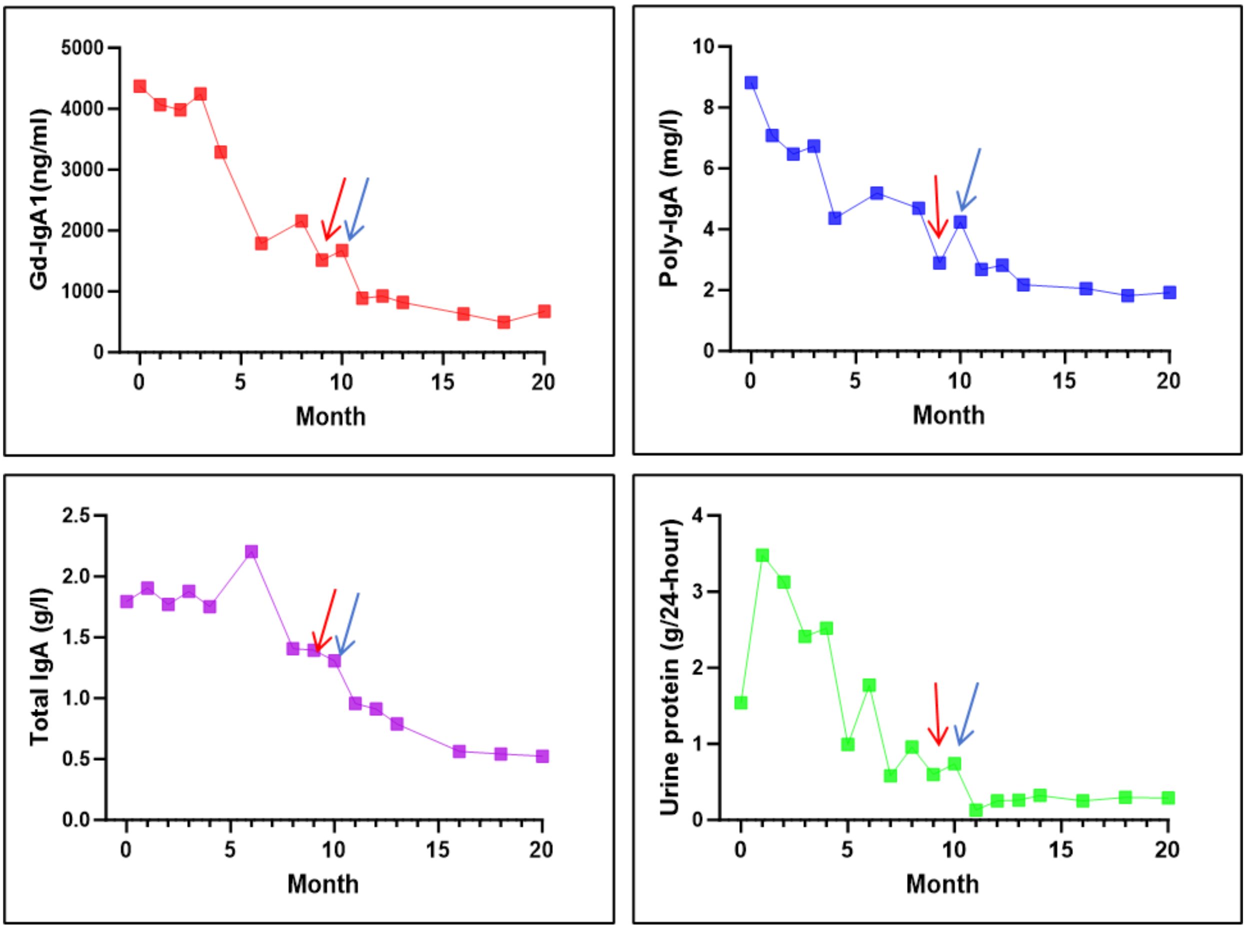

Supplemental Figure S2. Effects of Nefecon on (a) Gd-IgA1/total IgA and (b) poly-IgA/total IgA. Gd-IgA1, galactose-deficient IgA1.

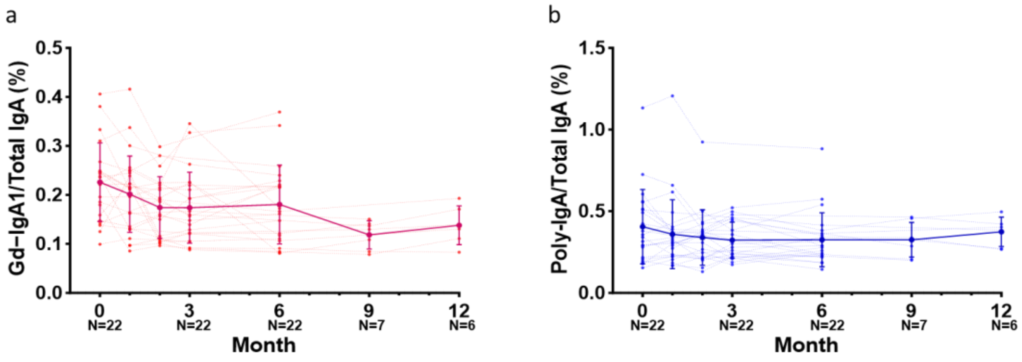

Gd-IgA1/Total IgA (SD), %

0.23	0.20	0.17	0.17	0.18	0.12	0.14
(0.08)	(0.08)	(0.06)	(0.07)	(0.08)	(0.03)	(0.04)

Poly-IgA/Total IgA (SD), %

0.41	0.36	0.34	0.32	0.33	0.33	0.37
(0.23)	(0.21)	(0.17)	(0.11)	(0.16)	(0.11)	(0.09)
